# Why do children with autism spectrum disorder have abnormal visual perception?

**DOI:** 10.3389/fpsyt.2023.1087122

**Published:** 2023-05-15

**Authors:** Rongyi Zhou, Xinyue Xie, Jiaojiao Wang, Bingxiang Ma, Xin Hao

**Affiliations:** ^1^Department of Pediatrics, The First Affiliated Hospital of Henan University of Chinese Medicine, Zhengzhou, China; ^2^Henan Provincial People's Hospital, Henan Institute of Ophthalmology, Zhengzhou, China; ^3^Renmin University of China, Beijing, China

**Keywords:** autism spectrum disorder, abnormal visual perception, visual cortex neurodevelopment, retina-lateral geniculate nucleus-visual cortex pathway, review and hypotheses

## Abstract

Autism spectrum disorder (ASD) is associated with severe impairment in social functioning. Visual information processing provides nonverbal cues that support social interactions. ASD children exhibit abnormalities in visual orientation, continuous visual exploration, and visual–spatial perception, causing social dysfunction, and mechanisms underlying these abnormalities remain unclear. Transmission of visual information depends on the retina-lateral geniculate nucleus–visual cortex pathway. In ASD, developmental abnormalities occur in rapid expansion of the visual cortex surface area with constant thickness during early life, causing abnormal transmission of the peak of the visual evoked potential (P100). We hypothesized that abnormal visual perception in ASD are related to the abnormal visual information transmission and abnormal development of visual cortex in early life, what’s more, explored the mechanisms of abnormal visual symptoms to provide suggestions for future research.

## Introduction

Autism spectrum disorder (ASD) is a lifelong neuro developmental disorder occurring in early childhood and is characterized by difficulties in communication and social interaction, restricted interests, repetitive behaviors, and sensory issues (1). Since firstly reported by Leo Kanner in 1943, it has evolved from a rare disease to a more common pediatric neuropsychiatric disease and has been of great concern to the global medical community (2, 3). The prevalence of ASD is estimated to have continued to increase, it has evolved from a narrowly defined, rare childhood disorder to a widely publicized, advocated, and researched long-term disorder that is considered fairly common and quite heterogeneous (4). The global prevalence of ASD was estimated to be approximately 1% in 2014 (2, 5), with a more recent review estimating the prevalence to be 1.5% in developed countries (6). ASD varies from mild to severe and dramatically affects personal and family quality of life. ASD represents a substantial economic burden, mainly due to the provision of support to adults who cannot function independently, and also involves higher health-care and school costs and loss of income for caregivers (7). It is not only a medical problem but also a significant social problem requiring greater attention that needs to be addressed medically.

Clinically, studies have reported that individuals with ASD not only display clinically impaired social interaction and behavior but also show significant differences in sensory and perceptual process functions (8), which was similar to Sensory Processing Disorders (SPD) (9). As a matter of fact, according to DSM-5, ASD is characterized by impaired social communication, restricted interests and repetitive behaviors, including the atypical sensory issues (10). SPD are a group of dysfunctions in modulating, organizing and using information from several sensory environment adaptation (11, 12). SPD can impact self-regulation, influencing behavior, daily-life activities, learning and neuropsychomotor development (13). Although SPD can be present also as isolated clinical conditions from early infancy to preschool age, their association with specific neurodevelopmental disorders is frequent. Particularly, the sensory processing dysfunction associated with specific social communicative deficits supports the ASD diagnosis (9, 10). Prospective studies have revealed that the diagnostic symptoms of ASD emerge during the latter part of the first and second year of life (14, 15). Differences in other developmental domains that are not necessarily specific to ASD, however, are detectable in the first year of life, including motor skills (16), attention to faces and social scenes, response to name, visual reception and visual orienting. Early in the second year of life, differences in language skills, and disengagement of visual attention (17) are also evident (18). Early diagnosis and intervention in ASD are advocated globally; Abnormal visual perception are listed as the primary symptom of “five no” behavioral markers in early clinical practice of ASD with strong evidence (19, 20). Individuals with autism have been characterized as “seeing the trees, but not the forest”: attuned to details of the perceptual world at the expense of the global percept they compose (21). Researchers reported that patients with ASD exhibited impaired ability to integrate socially relevant audiovisual information, and this contributes to the higher-order social and cognitive deficits in ASD (22). Abnormal visual perceptions appear early in children with ASD. Children who are finally diagnosed with ASD had only half the visual fixation time of healthy children at 24 months (23). Abnormal visual perception are considered important factors for early diagnosis of ASD, associated with social and cognitive impairments (24), and common in children with ASD (25). In summary, the main manifestations of abnormal visual perception in ASD are visual avoidance, visual exploration, and visual–spatial disorientation (26). Children with ASD begin to display the lack of or reduced visual fixation at an early age. Successful integration of visual inputs is crucial for both basic perceptual functions and higher-order processes related to social cognition (27, 28). Although there were a few studies and reports on abnormal visual perception in ASD, the mechanism remains unclear. Commonly, transmission of visual information depends on the retina-lateral geniculate nucleus (LGN)–visual cortex pathway. This process involves signal transmission, neurotransmitter release, visual information integration between retina, LGN, and visual cortex. The presence of abnormal visual perception in ASD children is easy to be widely recorded in clinic, and the evidence is conclusive (19, 20). We hypotheses that whether this may lead to abnormal transmission of visual information in retina-LGN-visual cortex pathway and determine the occurrence of abnormal visual perception symptoms of ASD? In this study, the main manifestations and underlying mechanisms of abnormal visual perception in ASD were reviewed. We formulated a hypothesis to provide guidance for the early diagnosis and research of ASD.

## Symptoms of abnormal visual perception in ASD

The sensory system is primarily responsible for the acquisition and processing of information from the external environmental and includes the visual, auditory, olfactory, and tactile systems (29). Visual information accounts for a large proportion of all external information obtained by organisms. Moreover, visual information is one of the most important channels for higher animals to obtain environmental information (30, 31). The visual information is processed by the nervous system and are eventually translated to higher-order social responses for organisms to respond appropriately to the environment. Many symptoms of abnormal visual information processing exist in children with ASD, including aimless visual exploration of a new environment (32); lack of visual fixation for meaningful social stimuli and difficulty diverting or disengaging vision (33); appear to read jumper, the opposite direction of writing, up and down stairs space sense abnormal visual space perception abnormality (34, 35). These abnormalities in visual acquisition are not conducive to normal social and cognitive performance (36). Combined with these abnormal manifestations, children with ASD commonly exhibit abnormalities regarding visual orientation (37), continuous visual exploration (38), and visual–spatial perception (35).

## Abnormal visual orientation

Children with ASD lack or reduce visual fixation to meaningful social stimuli and have difficulty diverting or disengaging their vision (39). This is believed to be a possible aberration of visual orientation. Visual orientation includes the involvement, diversion and disengagement of visual stimuli (40), which is defined as the process in which an individual disengages from a stimulus that has received visual attention, transfers attention from the previous stimulus, and then pays visual attention to the new stimulus (41). Numerous studies have demonstrated that patients with ASD are deficient in visual orientation (42). Bryson et al. (42) explored differences in visual attention shift and disengagement between infants at high risk for ASD (whose siblings were diagnosed with ASD) and typically developing (no family history of ASD) infants. The results revealed that high-risk infants with ASD had longer latency in visual attentional disengagement tasks and exhibited significant attentional disengagement difficulties, and when the stimulus was in the left visual field, these infants had significantly longer attentional disengagement latency. When the stimulus presented in the right visual field, the difference between the groups was not significant. The researchers believe that the left and right visual field asymmetry of attentional disengagement may be caused by functional deficit of the right hemisphere in individuals with ASD. Experiments with older children also confirmed that children with ASD responded longer in disengagement tasks and had deficits in visual attention disengagement, which leads to the detachment and transfer difficulty of local and global visual attention (43). Abnormal visual orientation in children with ASD have long been identified and studied. In 1943, Kanner summarized the visual characteristics of children with ASD as “they cannot perceive the whole unless they really pay attention to all the components of a thing.” (44). In the process of visual processing, normal people tend to find the connections among the components of visual objects in order to understand their overall meaning. Kanner and others (44, 45) have argued that people with ASD lack this holistic processing ability and tend to prioritize local features of stimuli. At present, there are two theories of “weak central coherence” and “enhanced perceptual functioning” for global and local abnormal visual orientation with ASD. The weak central coherence mentioned that individuals with ASD is not an intrinsic defect in overall processing, but the result of local or detailed processing advantages. Weak central coherence is not a cognitive defect, but a cognitive style. The theory could explain both some of the genius skills of people with ASD and some of their cognitive deficits. The weak central coherence theory suggests that abnormal global and local visual processing is related to abnormal neural connections (46). enhanced perceptual functioning theory proposed by Mottron and Burack (47) suggests that local processing advantages of ASD stem from lower perceptual processing. People with ASD have more advantages in information processing at the lower perceptual level than at the higher cognitive level, which will strengthen the memory of surface features of visual or auditory information, and thus affect the overall processing of information. This difference in cognitive processing between ASD and normal subjects is not a cognitive style, but is determined by their abnormal brain structure.

Visual orientation defects in patients with ASD are characterized by prolonged response time to achieve the transfer and separation of stimuli (17, 48), and it is even visually difficult to detach from the stimuli that have been noticed (43). Compared with the ability of visual transfer, the degree of impairment of visual attention disengagement ability in patients with ASD are more obvious (49). This often leads to patients persistently paying visual attention to one object, neglecting to collect other visual information and displaying symptoms of abnormal eye contact. The processing of visual orientation information is closely related to the prefrontal cortex, however, there is still academic controversy regarding the processing of visual–spatial working memory and visual orientation in different subregions of the prefrontal cortex. Some scholars believe that the ventral prefrontal cortex (VPFC) and the dorsolateral prefrontal cortex (DLPFC) have double separation in the working memory processing of visual objects and visual orientation information, and the VPFC is responsible for the working memory processing of visual objects, while the DLPFC is mainly responsible for the working memory processing of spatial information (50). However, some studies also believe that the VPFC is responsible for the information retention of objects and spaces, while the DLPFC may be more involved in the monitoring and manipulation of information (51). Defects in visual orientation appear within 12 months and continue into adulthood (52). Visual orientation is associated with the frontal and parietal lobes, but the exact mechanism is unclear (53).

## Abnormal visual exploration

Visual search is a complex cognitive process, which mainly comprises three stages, identifying and processing the basic characteristics of stimuli (such as color and orientation); paying attention to the planning and execution of assignments, and focusing processing on stimuli characteristics. Children with ASD often show symptoms of persistent and purposeless visual exploration of the strange environment (54). They appear to be looking for something all the time, and show increased visual interest in geometric figures, numbers, colors, and other objects (55). The advantage of visual search in individuals with ASD was first proposed by Plaisted, O’Riordan, and Baron-Cohen in 1998. Their study revealed that ASD has a higher accuracy rate when discriminating responses to unfamiliar objects (56). Subsequent research indicated that patients with ASD have the advantage of visual exploration, whether in the visual search test (57), response conflict test (58) or hidden figure task test (59). Patients with ASD displayed a stronger desire for visual exploration and response speed than healthy individuals. Some scholars purport that as a common symptom of abnormal visual perception in ASD patients, continuous visual exploration may be caused by the abnormal ability of visual information integration in ASD patients (60). Patients with ASD have difficulty combining pieces of information, so they are more likely to distinguish between objects by exploring local features. Other scholars believe that the occurrence of this symptom is related to the narrow or excessive focusing range of patients with ASD (61). Although a large number of studies have demonstrated that the symptoms of sustained visual exploration are related to the activation of the right superior parietal cortex, frontal operculum and occipital lobes (62), the causes and mechanisms of visual exploration symptoms remain unclear and require further exploration.

## Abnormal visual–spatial perception

Children with ASD often show off-line reading, writing backwards, writing out of bounds, abnormal spatial sense of objects, poor memory and other issues, which are considered to be associated with of visual–spatial perception abnormalities (63). Visual–spatial perception is the most dependent sensory function in human learning activities. Human beings transform visual–spatial perceptual information into visual–spatial working memory through the nervous system, and realize the temporary storage and processing of the visual perceptual information such as the color and shape of objects and spatial perception information of the object’s spatial location, which ensures visual–spatial orientation ability (64). Moreover, an individual’s ability to recognize faces and expressions is closely related to visual–spatial perception (65). In short-term memory, the high-precision storage of face feature information is crucial for the effective understanding, management and expression of emotions (66). Inadequate ability of patients with ASD to store short-term and high-precision face information is considered an important reason for the abnormal visual communication in patients with ASD (67).

## Visual information transmission and retina–LGN–visual cortex pathway

Abnormal visual perception including abnormal visual orientation, continuous visual exploration, and abnormal visual–spatial perception are common in patients with ASD, and these symptoms are confusing to clinicians and parents of children with ASD. Although experts have explored its pathogenesis from the perspectives of psychology (68), abnormal brain function (69), and abnormal neurotransmission (70); unfortunately, the pathogenesis of abnormal visual symptoms in ASD remains unclear.

In the latest diagnostic criteria, ASD is defined as a neurodevelopmental disorder, which results in the neurodevelopmental status of ASD receiving greater attention and research (71). Brain overgrowth in ASD has been widely documented, a more recent large-scale study has provided additional evidence for brain volume overgrowth between 12 and 24 months, and linked the rate of change in total brain volume during the second year of life to the severity of ASD-related social deficits (72). Importantly, research showed that cortical volume into cortical thickness and surface area to reveal that faster rates of cortical surface area growth from 6 to 12 months of age precedes brain overgrowth in the second year of life in infants who later developed ASD. The rate of surface area expansion from 6 to 12 months was also correlated with total brain volume at 24 months of age. These findings directly support the hypothesis generated from prior work that cortical hyper-expansion drives brain overgrowth in ASD (72). Brain overgrowth does not occur at birth, but in the latter part of the first year of life (73, 74); A more recent study demonstrated both accelerated rates of total cortical surface area expansion, and regionalized expansion in areas in the occipital, temporal, and frontal lobes in infants who later went on to develop ASD, with robust rates of expansion notable in the visual cortex (75). The severity of social deficits in ASD was associated with the rate of change in total brain volume in the second year of life (76). The visual cortex is the high nerve center of visual information processing and integration, and there is evidence that the overproduction of neurons alters neural connectivity, thereby affecting the function and behavior of the circuits (77). In patients with ASD, prolonged latency and decreased amplitude of P100 waves in visual evoked potentials have been widely recorded (78, 79). Brain activation and functional connectivity were investigated in high functioning autism using functional magnetic resonance imaging in an n-back working memory task involving photographic face stimuli. The autism group showed reliably lower activation in the inferior left prefrontal area. Which suggest that the neural circuitry of the brain for face processing in autism may be analyzing the features of the face more as objects and less in terms of their human significance. The functional connectivity results revealed that the abnormal fusiform activation was embedded in a larger context of smaller and less synchronized networks, particularly indicating lower functional connectivity with frontal areas (80). In general, the transmission of visual information is mainly through the retina–LGN–visual cortex pathway (81). The abnormal development of the visual cortex in the early stage of ASD suggests that there may be abnormalities in the transmission of visual information from retinal ganglion cells to the visual cortex. Whether abnormal visual information transmission from the retina to the visual cortex contributes to the formation of abnormal visual perception in ASD needs to be explored.

## Relationship and evidences of abnormal development of visual cortex and retinal–LGN–visual cortex neural circuits with abnormal visual perception

The acquisition of visual information mainly occurs in the retina (82), and further processing of information occurs in the retina, the LGN and the visual cortex (83). The retinal–LGN–visual cortex loop is the main method of visual information transmission and vision formation (84); therefore, research on the function of the visual system is mainly focused on these areas. Electrical activity is the most important information transmission carrier of the nervous system. The object can be seen because the reflected light of the object passes through the cornea, pupil, lens, vitreous of the eye and finally focuses on the retina to complete the light-electric signal conversion (85). The converted electrical signal passes are transmitted from the ganglion cells in the retina to the LGN in the brain, where they eventually reach the visual cortex for processing and integration to enable vision (86, 87). There is an obvious association between abnormal visual perception and the retina–LGN–visual cortex neural circuits.

The retina is the first station for receiving visual information and the only source of visual information in higher animals (88). The retina is divided into 10 anatomical layers from the outer layer to the inner layer. The outermost layer is the pigment epithelium and the inner layer is the inner boundary layer (89). Different layers are composed of neurons with varying functions. Photoreceptor cells are responsible for photoelectric conversion of stimulated light into a hyperpolarization potential on the cell membrane, and subsequently transmitting the synapse signals to bipolar cells. The signals are processed by the bipolar cells and relayed to the ganglion cells through the synapses. The ganglion cells encode the visual information processed by the retina into nerve impulses and then transmit it to the brain (85). This transmission process is regulated and processed by both horizontal and amacrine cells, and finally the different elements of visual information, such as brightness, color, motion speed and direction are transmitted to retinal ganglion cells, which are the last station of visual information in the retina (85, 90). The retinal ganglion cells process and integrate the information and then transmit the electrical signals to the relay cells in the genu outside the lower brain region. Although the function of the retina is extremely complex, the basic function of the retina is photoelectric signal conversion of visual information and simple processing of different elements of visual information, such as color and brightness, and ensuring that the information is transmitted to the LGN *via* ganglion cells in a hierarchical manner (91). The visual color preference in children with autism may result from the abnormal processing of visual elements in the retina.

The LGN is located on either side of the thalamus and is one of many sensory relay nuclei in the thalamus (92). Anatomically, the LGN is the first to sixth layer from bottom to top. Various types of lateral geniculate cells are distributed in different layers, receiving input from different subtypes of retinal ganglion cells, and then transmitting this information to the visual cortex (93). The projection of retinal ganglion cells to various layers of the lateral geniculate is regular (94, 95). Each layer of the lateral geniculate forms a certain retinal projection relationship with the corresponding half of the retina in the contralateral field of vision, that is, when the adjacent areas of the retina are projected to the lateral geniculate, they are also adjacent or overlapping. This is important because spatial location information can be retained in the process of visual information transmission (96–98). Is the abnormal visual–spatial perception and visual orientation in children with autism related to the disorder of the layering of retinal ganglion cells’ visual information to the LGN? In addition, the LGN is considered as a transfer station for visual information. On the one hand, it receives information from retinal ganglion cells and transmits it to the visual cortex and, on the other hand, receives feedback information from the visual cortex and regulates the electrical activity of visual neurons through neurotransmitters to achieve a balanced effect in visual information processing (99, 100). The balance of excitatory and inhibitory transmitters plays a fundamental role in the normal function of all levels of cells in the retina and LGN and the normal transmission of visual information (101, 102). An imbalance between excitatory/inhibitory neurotransmission has been posited as a central characteristic of the neurobiology of autism (103). Distinct changes in the neurochemical composition, functional architecture and signaling fidelity of early visual cortex are observed in autism. The excitatory-inhibitory imbalance hypothesis postulates dysregulation of the gamma-aminobutyric acid (GABA) and glutamate (Glu) neurotransmitter systems as a common underlying deficit in individuals with autism spectrum disorders (104). Specifically, magnetic resonance spectroscopy (MRS) is a noninvasive imaging technique that generates a frequency spectrum by exploiting the nuclear magnetic resonance properties of hydrogen atoms. Metabolites can be identified by the position of their signal peak. MRS detect implicated that GABA has influence in visual suppression deficits in autism. Researches showed that the level of one, gamma-aminobutyric acid, in the visual cortex was directly related to search abilities in children with ASD (105). ASD exhibited elevated levels of the inhibitory neurotransmitter GABA in the left dorsolateral prefrontal cortex (106). These findings support the theory of an imbalance between excitatory and inhibitory equilibrium in patients with autism spectrum disorders.

The visual cortex is the senior center of the visual information processing and mainly refers to the part of the cerebral cortex that is primarily responsible for processing visual information. It is located in the occipital lobe at the back of the brain, including the primary visual cortex [also known as the striate cortex or visual first area (V1)] and grain outside surface (such as the second, third, fourth, and fifth visual areas, V2, V3, V4, V5) (107), and the primary visual cortex receives information from the LGN, which is then transmitted *via* V2 and V3 to V4, V5, and higher brain regions. The orderly layering of cells is a common structural feature in the retina, LGN, and visual cortex (108). Many cells with the same characteristics are arranged spatially in the visual cortex according to certain rules. This functional structure of the cortex, namely the functional construction of the cortex, presents a columnar distribution along different levels of the cortex, such as directional, azimuthal, ocular dominant, spatial frequency, and color columns (109–111). The formation of this structure plays an important role in the processing of sensory information in the cortex. The function of the visual cortex is complex, and the specific form of processing and integration of visual information by the visual cortex has not been elucidated. We have observed a clear phenomenon of rapid development of the visual cortex area while constant thickness in early development of children with ASD (72, 75). Nassi and Callaway (112) clearly laid out the projections of the major retinal ganglion cell (RGC) types, with their photoreceptoral sources as well as termination patterns in the lateral geniculate nucleus (LGN) and their projections into primary visual cortex (V1) and onwards into the dorsal and ventral cortical streams. In a disruption to conventional views suggesting that LGN-V1 connections drive development of both cortical streams it now appears that the dorsal stream with its MT projections precedes development of the ventral stream. Marmoset studies demonstrate that Dorsal stream associated areas were also found to emerge significantly earlier than ventral stream areas (113, 114).The functional development of each stream has shown a somewhat variable pattern in both human and non-human species. Some argue that when compared to the ventral stream, dorsal stream related areas mature later, are less activated and increase more in volume (115–117) while others find an earlier dorsal stream maturational pattern, occurring at 4–5 (118) and 6 years old (119). Parallel development and maturation of both streams occur at somewhat similar times in macaques aged from around 1 month to 2 years (120). By comparison, human visual maturation appears to occur from 3 to 12 years. Several factors are at play here. For example, the maturation of visual spatial acuity depends on the maturation of the photoreceptor outer segments (121), eyeball axial length (affecting photoreceptor spacing, hence angular subtense), as well as cortical development and maturation including the processes of myelination (122). It is reasonable to speculate that the development of visual cortex hierarchy is overexpanded in children with ASD and could potentially lead to abnormal visual information processing.

## Hypothesis of the correlation among ASD abnormal visual perception and abnormal visual information transmission and abnormal development of visual cortex in early life

The visual system includes the entire system from the retina to the LGN of the thalamus to the cerebral cortex. Therefore, all matters regarding visual information are related to the visual system (123). The retina is the core component of the visual function of the eye, and it is responsible for converting the optical signals of the visual world into electrical signals to the brain. The ganglion cells in the retina can collect and transmit the light intensity and color information (124, 125). More importantly, ganglionic cells can sensitively detect spatial light and dark contrast and color contrast through the central-peripheral concentric circle against the visual receptive field (126, 127), which forms the physical basis of shape vision (128). Face recognition abnormality is one of the most typical features of ASD with abnormal visual perception and difficulties in social communication (129–131). It is mainly manifested in avoidance of sight, long reaction time of face recognition, and low accuracy of face recognition (132, 133). These problems cause patients with ASD to be unable to correctly and quickly understand and judge the expressions or emotions of others, which seriously hinders their participation in normal social interaction activities (134). The face has rich visual contrast information; studies have shown that the lack of visual attention to the face in children with ASD may be related to the abnormal processing of facial light–dark contrast and color contrast information (135–137). In addition, when the brain processes visual information, it is not a single brain area that performs functional activities, and multiple brain areas are involved in this process. Fusiform gyrus (FFG) is a specific area of the brain’s higher visual cortex for face recognition, which is more sensitive to face stimuli (138). Kleinhans et al. (139) found that when children with ASD were dealing with face stimuli, the activation of the FFG was reduced. A large number of retrospective studies has confirmed that face recognition dysfunction is common in ASD patients, manifested as lack of attention to human faces, abnormal understanding of facial expressions, and abnormal face processing strategies (130). The abnormal connection of BA17 to bilateral FFG reconfirmed that children with ASD may experience face recognition impairment due to insufficient FFG activation when processing face-related visual information.

**Figure 1:**
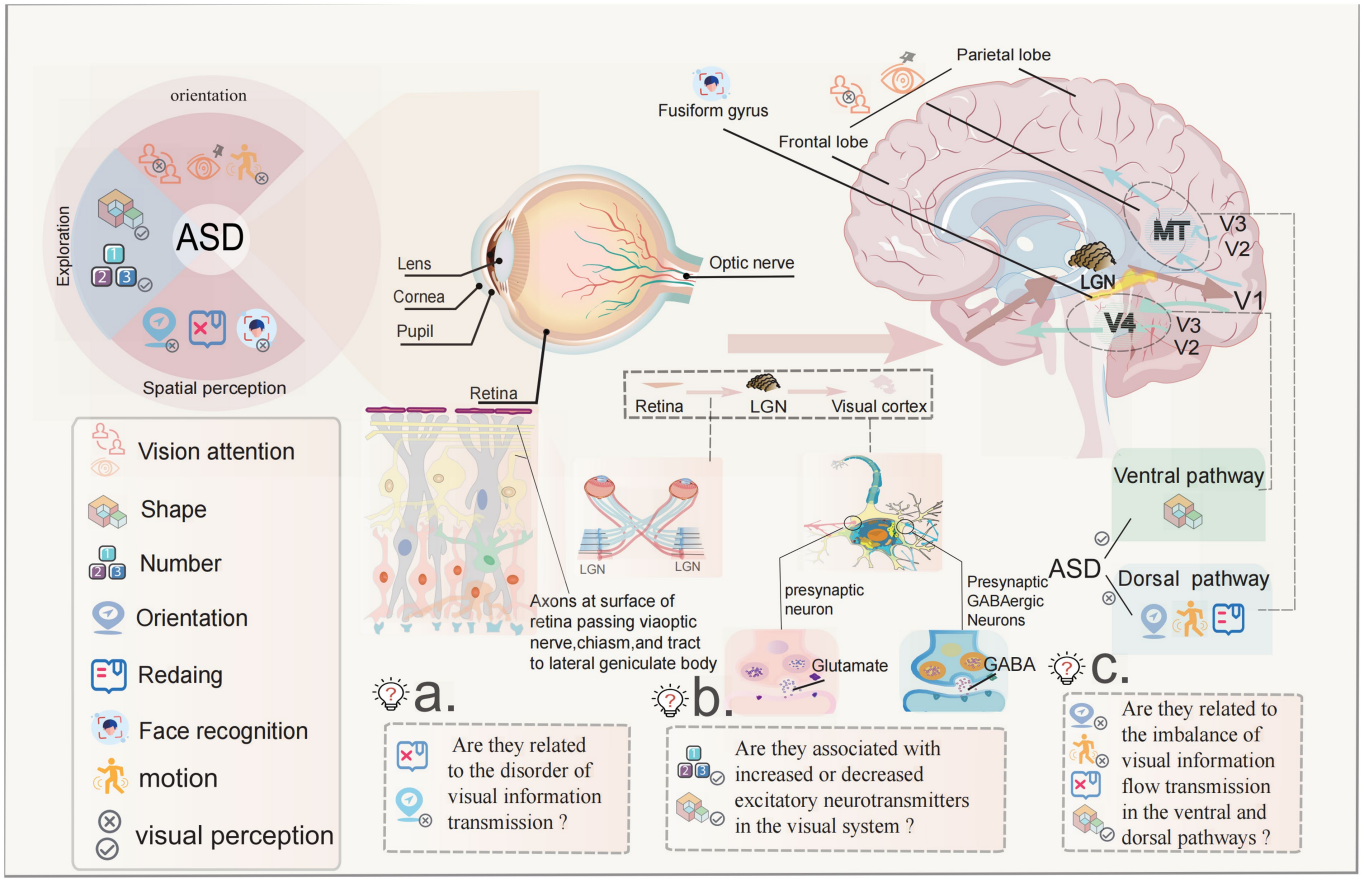
The potential pathogenesis of abnormal visual perception in ASD children with ASD have abnormal visual perception as visual avoidance, visual exploration, and visual–spatial disorientation, which are listed as the “five no” behavioral markers in early clinical practice with strong evidence. ASD is defined as a neurodevelopmental disorder, studies have found that children with ASD have rapid development of the visual cortex between the ages of 1–2 years, as evidenced by rapid expansion of cortical area and slow changes in thickness. Is this related to the rapid expansion of the visual cortex? The transmission of visual information is accomplished following the sequence of retina-LGN-visual cortex, the balance of corresponding signaling between the layers is the key to the preservation of visual–spatial location information. Is the abnormality of visual–spatial perceptual information related to the disturbance of the level of visual information transmission? The FFG of visual cortex is the core area of face and facial expression recognition. There are functional abnormalities in this area in ASD children. Is this the cause of abnormal face recognition in ASD children? There is a transmitter balance between excitement and inhibition in the transmission of visual information. Studies have shown that there is an imbalance in children with ASD. Is there a relationship between the enhancement and weakening of excitatory transmitters and the symptoms of continuous visual exploration? In the visual cortex, there is the transmission of ventral flow and dorsal flow signals when processing visual information. The ventral pathway is good at shape perception, the dorsal pathway is good at spatial position perception, and most ASD children are good at shape perception while visual–spatial perception is abnormal. Is there a correlation between them?

We previously proposed that children with ASD have abnormal visual–spatial perception. In visual information transmission, the level correspondence between the retina and the LGN is the key to preservation of visual–spatial position information. The abnormal visual–spatial perception information is related to visual information and the disorder transmission level. At the same time, the visual cortex also affects visual orientation and visual–spatial perception functions, as well as final processing of color and contrast information (140). A distinctive feature of most primary visual cortex cells is that they have strong orientation selectivity, creating the final sense of direction and space (141, 142). The LGN serves as a transfer station for visual information transmission, which is responsible for the two-way communication of information between the retina and the visual cortex and maintains the balance of information transmission. The signal transmission between cores in the brain is mainly conducted in the form of chemical signal transmission. The regulator of the LGN depends on the dynamic balance of the visual cortex and retinal excitatory and inhibitory neurotransmitters, such as glutamate, gamma aminobutyric acid and cholinergic transmitters and their receptors, to affect the strength of visual function (143). As early as 2001, Hussman (144) proposed the theory of neurotransmitter imbalance in ASD and found that the imbalance of excitatory Glu neurotransmitter and inhibitory Gabaergic neurotransmitter system was closely related to the incidence of ASD. As an ion channel inhibitor, Bumetanide can restore the excitatory GABA in the immature or damaged brain to inhibitory effect by inhibiting the influx of chloride ions, so that it can be used in the treatment of ASD (145, 146). Studies have shown that the down-regulation of the expression of excitatory nerve cell receptors in the visual cortex is closely related to weakened visual function (147, 148). Hence, it is feasible to speculate that the increase of excitatory transmitters in the visual system and the appearance of visual exploration symptoms, as well as the increase of inhibitory transmitters may be related to the decrease in visual fixation and the weakened visual orientation in ASD. The hypotheses is presented in Figure 1.

Vision should be considered a prerequisite for neurodevelopment as a whole, for the evolution of motor abilities and learning, for a childs neuropsychological and psychic development, and for his emotional and affective growth (149). Vision is necessary from the beginning of life to create a relationship with caregivers through eye-contact, to develop preverbal communication, to structure cognitive, motor, affective, and social intentionality and reciprocity (150, 151). Visual inputs is crucial for both basic perceptual functions and higher-order processes related to social cognition. Decades of brain imaging research on the neural underpinnings of ASD strongly suggest that autism is not a strictly localized brain disorder, but rather a disorder involving multiple disordered brain connectivity functional neural networks (152, 153). Based on the clinical symptoms of abnormal vision perception in children with ASD and the abnormal development of the visual cortex, we speculate that the occurrence of abnormal visual symptoms are related to the abnormal visual information transmission and abnormal development of visual cortex in early life. Exploring the mechanism of abnormal visual perception in children with ASD will not only help to solve the core symptoms of ASD, but also greatly help to improve the social adaptability of children with ASD. At the same time, this research will be beneficial to improve the development level of all children with visual impairment (149). At present, the evidence of abnormal visual cortex development in children with ASD before 2 years of age has been fully found, but the mechanism of its influence on visual information transmission has not been proposed and elucidated. This provides a new research direction for us to explore the mechanism of abnormal visual symptoms in ASD, and it is hoped that this hypothesis will be explored in the future. Abnormal visual perception in ASD This line of research indicating the benefits of developmental facilitation for children with sensory disorders. Further welldesigned and larger experimental studies are needed to strengthen the generalizability of the findings and their use in early interventions for children with neurodevelopmental disability.

## Data availability statement

The original contributions presented in the study are included in the article/supplementary material, further inquiries can be directed to the corresponding author.

## Author contributions

RZ drafted the manuscript. BM offered professional guidance to the paper. JW provides guidance on ophthalmology. XX participated in drawing picture, article and reference revision, and put forward important ideas for article revision. XH contributed to opinion offer to the paper. All authors contributed to the article and approved the submitted version.

## Funding

This research was funded by National Natural Science Foundation of China Youth Fund (82104928) and General Program of China Postdoctoral Fund (No. 2021M701123).

## Conflict of interest

The authors declare that the research was conducted in the absence of any commercial or financial relationships that could be construed as a potential conflict of interest.

## Publisher’s note

All claims expressed in this article are solely those of the authors and do not necessarily represent those of their affiliated organizations, or those of the publisher, the editors and the reviewers. Any product that may be evaluated in this article, or claim that may be made by its manufacturer, is not guaranteed or endorsed by the publisher.
